# How Dermatologists Can Aid Nondermatologic Professionals Using the Figure 1 App: Case Analysis

**DOI:** 10.2196/60500

**Published:** 2024-11-13

**Authors:** Alexis Coican, Nathaniel A Marroquin, Alexa Carboni, Sara Holt, Morgan Zueger

**Affiliations:** 1 Department of Graduate Medical Education, Hospital Corporation of America (HCA) Orange Park Medical Center, Orange Park, FL, United States; 2 College of Osteopathic Medicine, Rocky Vista University, Parker, CO, United States; 3 Department of Dermatology, Largo Medical Center, Largo, FL, United States

**Keywords:** dermatology, app, nondermatologic professional, dermatologist, nondermatologist, mHealth, health professional, medical education, social media, treatment, diagnostic

## Abstract

We found that third-party apps such as *Figure 1* were used predominantly by nondermatologist medical personnel for collaboration in medical decision-making related to dermatologic conditions conducted with other health care providers. This finding calls attention to the need for more readily available resources for nondermatologist health care providers encountering patients with skin-related conditions, with the added benefits of trained dermatologists being allies on such medical app platforms, and the platform allowing for recognition of instances when additional consultation or referral to trained dermatologists for more complex cases is appropriate.

## Introduction

*Figure 1* is a mobile app in which thousands of health professionals share and discuss medical cases in real time. *Figure 1* provides a platform for medical education and collaboration and is available in close to 100 countries. The app works in a similar way to Facebook or Instagram; images are posted with captions consisting of relevant patient information, and other users offer diagnostic and treatment advice by posting in the comments section. Social media is a ubiquitous part of modern life, and the use of apps like *Figure 1* to obtain medical knowledge and insight has become very popular [1]. Based on a study conducted by Ranpariya et al [2], only 4% of dermatologic content on social media is produced by board-certified dermatologists. Therefore, the purpose of this letter is to analyze content within the *Figure 1* app in regard to diagnostic agreement with nondermatologic health care professionals and discuss the advantageous role dermatologists can play in the use of the app in daily medicine.

## Methods

### Recruitment

We collected and examined 300 dermatologic cases posted on the main feed of the *Figure 1* app between June 2023 and August 2023 using Microsoft Excel. The data were organized and totals were calculated for the specialties of the authors and their reasons for posting. This research project did not involve human subjects as defined by US federal regulations under 45 CFR 46.102(f). The study used publicly available data from social media platforms that did not include any personally identifiable information or private data that would require informed consent or institutional review board approval.

### Statistical Analysis

Aggregate data for both health care provider training and role (ie, physician, nurse practitioner, physician assistant, or registered nurse) and specialty training were cumulatively added and represented as percentages of the total 300 dermatologic cases.

## Results

Of the 300 cases analyzed, 151 were presented by nondermatologic physicians, 8 were presented by dermatologists, and the remaining 141 were presented by nurse practitioners, physician assistants, and registered nurses, with no specialty indicated. The specialties most represented among the 151 cases presented by nondermatologic physicians were internal medicine (n=65, 43%), family medicine (n=53, 35%), and emergency medicine (n=33, 22%). All cases were seeking assistance in rash or lesion identification and for future treatment.

## Discussion

### Principal Findings

Of the 300 cases assessed, 292 were presented by nondermatologic health care professionals seeking further assistance in rash and lesion identification. The data we gathered from this app prompted us to consider if primary care physicians (PCPs) and nondermatologic health professionals are receiving adequate education regarding appropriate treatment and criteria for common cutaneous ailments for referral.

### Comparison With Prior Work

In a study conducted by Patro et al [3], the overall agreement between diagnoses made by a PCP and a dermatologist was 56%, with poor diagnostic agreement seen most in psoriasis and eczema. If dermatologists and nondermatologic health professionals can only agree 56% of the time, and only 8 of the 300 cases (2.7%) were posted by a dermatologist, as seen in *Figure 1*, then there is a concern related to inaccuracies and the spread of misinformation.

### Limitations

Reporting errors among *Figure 1* app users should be considered when stating the health care provider role and case presentation. The strengths of this study could be further developed by increasing the sample size and lengthening the time of data collection, and these should be considered in future research.

### Conclusion

PCPs are usually a patient’s first contact regarding their health. Thus, PCPs have a unique opportunity to recognize and treat common dermatological diseases, including benign skin lesions, fungal infections, acne, and atopic dermatitis. Knowledge and skills training should be equipped to prepare PCPs for management of certain conditions, such as skin cancer, due to their impact. Additionally, it is important for users to be aware that virtual consultations such as these should not take the place of a formal evaluation by a board-certified dermatologist. However, the larger number of cases that were presented by health care professionals without formal dermatologic training reveals a gap that can be filled by trained dermatologists. Dermatologists can use *Figure 1* to aid trained professionals who are not dermatologists by providing knowledge, bridging diagnostic gaps regarding common cutaneous conditions, and preventing the spread of misinformation and misdiagnoses pertaining to the skin.

## Abbreviations

PCP: primary care physician
